# Volatiles released by undamaged plants mediate the adaptive growth strategies in neighbors

**DOI:** 10.1093/jxb/erag252

**Published:** 2026-05-28

**Authors:** André Åbonde, Merlin Rensing, Jannicke Gallinger, Vasti Thamara Juárez-González, Iris Dahlin, Dimitrije Markovic, German Martinez, Velemir Ninkovic

**Affiliations:** Department of Ecology, Swedish University of Agricultural Sciences (SLU), Uppsala 75007, Sweden; Department of Ecology, Swedish University of Agricultural Sciences (SLU), Uppsala 75007, Sweden; Department of Ecology, Swedish University of Agricultural Sciences (SLU), Uppsala 75007, Sweden; Federal Research Centre for Cultivated Plants, Institute for Plant Protection in Fruit Crops and Viticulture, D-69221 Dossenheim, Germany; Department of Plant Biology, Uppsala BioCenter, Swedish University of Agricultural Sciences (SLU) and Linnean Center for Plant Biology, Uppsala 75007, Sweden; Department of Crop Production Ecology, Swedish University of Agricultural Sciences (SLU), Uppsala 75007, Sweden; Department of Ecology, Swedish University of Agricultural Sciences (SLU), Uppsala 75007, Sweden; Department of Plant Biology, Uppsala BioCenter, Swedish University of Agricultural Sciences (SLU) and Linnean Center for Plant Biology, Uppsala 75007, Sweden; Department of Ecology, Swedish University of Agricultural Sciences (SLU), Uppsala 75007, Sweden; The James Hutton Institute, UK

**Keywords:** Constitutive VOCs, growth–defense trade-off, plant volatile interactions, plant–plant communication, transcriptional response, volatile organic compounds

## Abstract

Plants continuously emit volatile organic compounds (VOCs), which can influence the physiology and behavior of neighboring plants. While the ecological role of stress-induced VOCs is well established, the function of constitutive VOCs released by undamaged plants in mediating plant–plant interactions remains less understood. Here, we demonstrate that barley plants can detect the growth rate of undamaged conspecific neighbors through constitutive VOCs and respond by modulating their growth–defense trade-off accordingly. Exposure to volatiles from cultivars with contrasting growth (slow or fast) triggered distinct shifts in biomass accumulation and gene expression in receiver plants, whereas VOCs from cultivars with similar growth rates had negligible effects. Transcriptomic analysis revealed cultivar-specific transcriptional reprogramming of growth- and defense-related pathways, suggesting that constitutive VOCs convey information about emitter identity and competitive vigor that receiver plants use to adaptively reallocate resources and prime stress responses in anticipation of competition. These findings uncover a previously unrecognized role for constitutive VOCs as reliable cues of emitter identity and vigor, mediating adaptive responses in neighboring plants under competitive scenarios.

## Teaser

Plants detect growth rates of their neighbors through constitutive volatile cues and adapt their growth–defense trade-offs, revealing that plants use volatile cues to assess competition before it begins.

## Introduction

Throughout their life cycle, plants continuously perceive and respond to environmental signals, adjusting their growth and development in response to prevailing conditions (Pieterse *et al.*, 2012; Lamers *et al.*, 2020). Among these signals, volatile organic compounds (VOCs) mediate interactions not only with herbivores, pathogens, and natural enemies, but also with neighboring plants (Bouwmeester *et al.*, 2019). These VOCs provide a chemical ‘fingerprint’ of the physiological state of an emitter, enabling neighboring plants to assess the growth dynamics and stress status of the emitter (Effah *et al.*, 2019; Ninkovic *et al.*, 2021). Most research has focused on stress-induced VOCs, released upon herbivore or pathogen attack, which have been shown to trigger defense responses in neighboring plants (Das *et al.*, 2013; Bouwmeester *et al.*, 2019; Brosset and Blande, 2022; Murali-Baskaran *et al.*, 2022). In contrast, the ecological functions of constitutive VOCs emitted by undamaged plants are far less studied. Emerging evidence shows that these constitutive VOCs encode genotype-specific information that plants can use to distinguish between conspecific neighbors (Ninkovic *et al*., 2016; Dahlin *et al*., 2018). Exposure to constitutive VOCs can induce resistance in neighboring plants, making them less attractive to herbivores (Kheam *et al.*, 2023; Markovic *et al.*, 2025) and more attractive to natural enemies (Ninkovic and Pettersson, 2003; Ninkovic *et al.*, 2011, 2016), and may also alter their biomass allocation (Ninkovic, 2003). Whether constitutive VOCs more broadly modulate fundamental plant processes in neighboring plants, such as growth and competitive interactions, remains less understood and largely unexplored (Brosset and Blande, 2022).

Stress-induced VOCs not only mediate interactions with other organisms but also play a central role in regulating physiological processes within the plant, including defense activation and resource allocation in neighboring plants (Brosset and Blande, 2022). Plant defense strategies encompass both constitutive defenses, maintained at baseline levels, and inducible defenses, which are activated upon detection of stress signals. Induced responses in plants can be triggered by mechanical or chemical signals and are orchestrated through key phytohormonal pathways, including jasmonic acid, salicylic acid, and ethylene signaling. These pathways coordinate transcriptional reprogramming that leads to the activation of targeted defense response (Erb *et al.*, 2012). Such defenses can involve biosynthesis of secondary metabolites, production of defensive proteins, structural reinforcements such as cell wall thickening, and the emission of herbivore-induced plant volatiles (HIPVs). HIPVs serve dual ecological functions by both attracting natural enemies of herbivores and priming defense responses in neighboring undamaged plants (War *et al.*, 2012). These complex responses are regulated through the integration of external cues, such as tissue damage or VOCs from neighboring plants, and internal hormonal signaling pathways (Lamers *et al.*, 2020).

The biosynthesis of defensive metabolites requires significant metabolic investment, diverting resources away from growth and reproduction, and continuously challenging plants to balance resource allocation between defense and growth (Huot *et al.*, 2014; Züst and Agrawal, 2017). In stressful environments, the activation of defense-related pathways and their associated transcriptional reprogramming often leads to growth suppression, while in favorable conditions, plants tend to prioritize growth and development over defense investment (He *et al.*, 2022). However, growth–defense trade-offs are not always expressed, making it important to determine whether they occur in the specific system (Züst and Agrawal, 2017). Gaining mechanistic insight into how transcriptional networks coordinate those opposing demands is essential for understanding the physiological trade-offs plants make under varying environmental conditions. Genome-wide transcriptomic analyses, enabled with high-throughput sequencing technologies, offer powerful tools to dissect these resource allocation decisions by identifying genes activated under specific conditions (Upton *et al.*, 2023). However, despite increasing interest in volatile-mediated plant–plant communication, the transcriptional responses triggered specifically by constitutive VOCs remain largely uncharacterized.

**Fig. 1.**
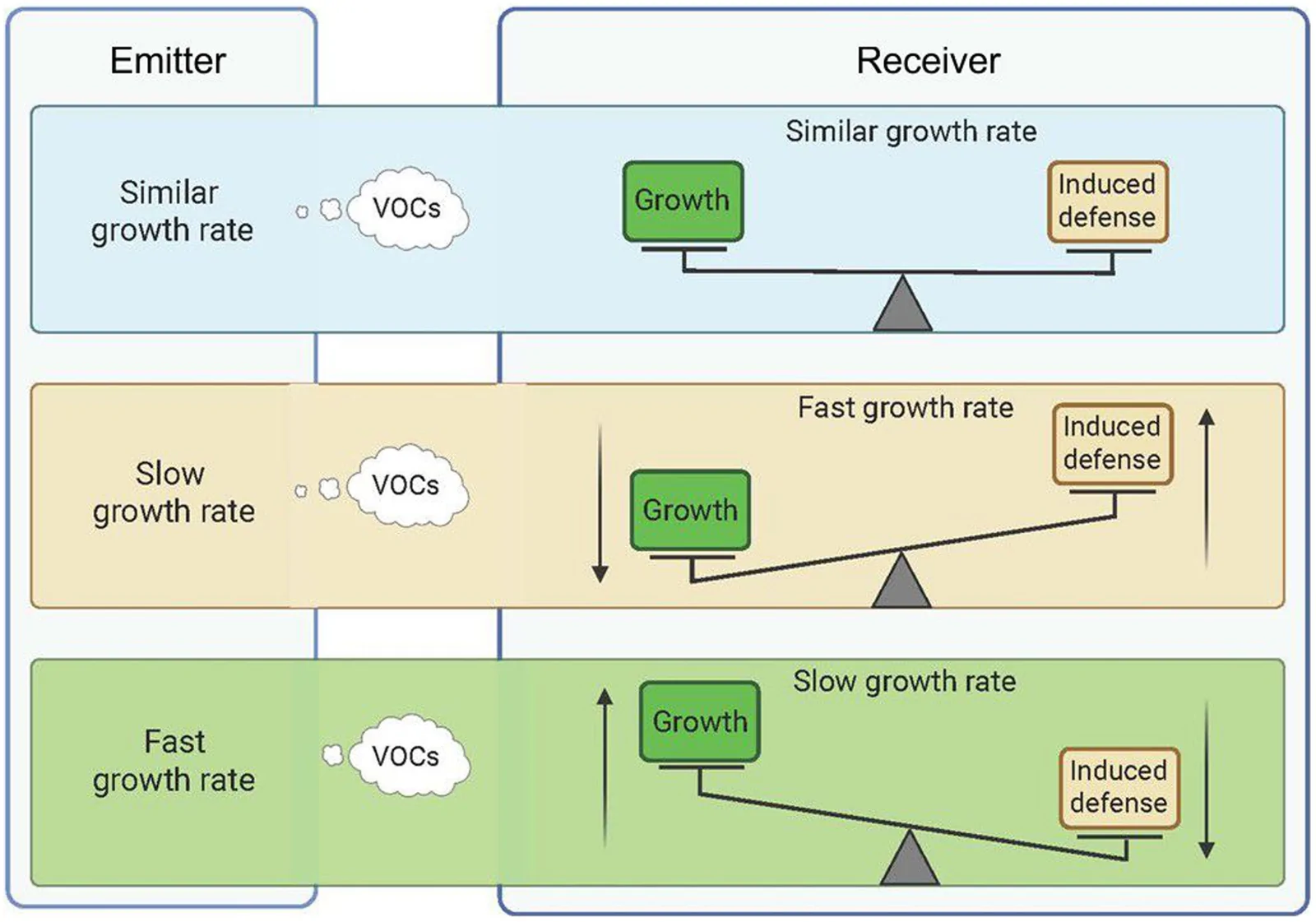
Conceptual framework illustrating our hypothesis. Constitutive VOCs emitted by undamaged emitter plants mediate adaptive growth strategies in neighboring receiver plants. Receivers with growth rates similar to the emitter remain unaffected. Fast-growing receivers reduce growth and increase investment in induced defenses when exposed to VOCs from slow-growing emitters. In contrast, slow-growing receivers increase growth at the expense of induced defenses when exposed to VOCs from fast-growing emitters. Created with BioRender. Åbonde, A. (2026) https://BioRender.com/sgp8ljw.

Here, we test the hypothesis that plants can perceive and respond to the growth strategies of undamaged conspecific neighbors through their constitutive VOC profiles (Fig. 1). We focused on three spring barley (*Hordeum vulgare*) cultivars with contrasting growth rates (slow, intermediate, and fast) as a proof-of-concept system for evaluating this hypothesis, each previously shown to differ in VOC-induced defense against aphids. Notably, VOCs emitted by certain undamaged cultivars have been shown to disrupt aphid olfactory preferences in laboratory assays and reduce aphid infestations under field conditions (Dahlin *et al.*, 2018; Kheam *et al.*, 2023). Whereas previous studies have primarily emphasized VOC-mediated defense responses, our study investigates the complementary aspect of the growth–defense trade-off by quantifying biomass production and genome-wide gene expression in VOC-exposed plants.

## Materials and methods

### Plant materials

Spring barley (*H. vulgare* L.) cultivars Fairytale, Luhkas, and Salome (Scandinavian Seeds AB, Sweden) were selected based on their different growth strategies, previously characterized by slow, intermediate, and fast growth rates, and representing genetically distinct pedigrees developed by breeding programs across different countries (Dahlin *et al.*, 2020). Fairytale and Luhkas are fodder-type cultivars, while Salome is a malt-type cultivar. Fairytale [pedigree: Colston ×(Recept×Power)] was developed by Sejet Plant Breeding, Denmark, Luhkas (pedigree: Annabell×Prestige) by R.A.G.T seeds Ltd, UK, and Salome [pedigree: Auriga×(Publican×Beatrix)] by Nordsaat Saatzucht GmbH, Germany (Dahlin *et al*., 2020). All three cultivars have been shown to produce distinct constitutive VOC profiles (Dahlin *et al*., 2018).

### Growing conditions

The climate in the growth chamber was maintained at 20±2 °C, with a light regime of 16 h of light and 8 h of darkness (L16:D8), and 60% relative humidity. Light was provided by Light4Food lamps, situated 1 m above the table, with a central light strip (Philips LED, 77 W, Poland) and side lights located to its left and right (Philips LED lighting IBRS, 55–60 W, the Netherlands), each following their own light regimen. Plants were grown in perforated plastic tubes (90×5 cm) filled with clean sand (Silversand 55, Sibelco Europe, Mölndal, Sweden). These tubes were positioned beneath the bench, with the open end of each tube passed through a 5×2 cm hole in the bench and secured with a staple (see Supplementary Fig. S1 for the experimental setup). The lower end of each tube featured two drainage holes, each 3 mm in diameter, which were connected to drainage pipes. To ensure darkness for the roots, a black plastic curtain was placed around the table, and a foam plastic plug was used to seal the hole, thereby blocking light and limiting airflow from beneath.

Two days before sowing, the tubes were connected to a watering drip-irrigation system to ensure adequate sand moisture for plant growth. Seeds were germinated on wet filter papers in Petri dishes at room temperature (20 °C) in a dark room for 2 d. Uniform sized pre-germinated seeds were selected, and a single seed was planted at a depth of 1 cm in the sand of each tube. A commercial plant nutrition solution (Wallco Växtnäring, Sweden) was delivered via an automatic watering system with a dosage of 2 ml l^–1^ on a fixed schedule, ensuring a controlled water and nutrient regime throughout the experiment.

### Plant exposure to the volatiles from another cultivar

Six days after the initial sowing, individual plants in the growth stage of Zadoks (Zadoks *et al.*, 1974) decimal code 10–11 (first leaf unfolding to second leaf visible) were enclosed using the transparent twin-chamber cages, as shown in Supplementary Fig. S1. At this stage, treatment combinations were introduced in the twin chamber to initiate VOC exposure throughout the growth period. Plants were already arranged to minimize mutual shading, and the transparent Perspex cages ensured uniform light conditions across all replicates. The Plexiglas (acrylic glass) walls prevent reflections while allowing full transmission of overhead light, ensuring that differences in biomass could not be attributed to shading or unequal light exposure. These cages, placed on the table within the growth chamber, ensured the spatial separation and the containment of paired plants, while enabling the exposure of one cultivar to volatiles emitted by another cultivar. The system comprises a series of Perspex twin-chamber cages (10×10×80 cm), and each cage serves as a replicate. According to Ninkovic (2003), air was introduced into one chamber, designated as the inducing chamber (IC), through an opening (0.7 cm Ø), and then passed through an opening (0.7 cm Ø) in the partition wall into the adjacent responding chamber (RC). The air from the RC was directed into a vacuum tank before being vented outside the growing chamber. This design ensured that only above-ground volatiles were exchanged between the chambers, thereby excluding any influence of below-ground compounds. The airflow through the system was 1.3 l min^−1^.

### Experimental treatments

Two separate experiments were conducted, each using a different receiver cultivar: slow-growing cultivar Fairytale in the first experiment and fast-growing cultivar Salome in the second. In the Fairytale experiment, the treatments were: Fairytale exposed to Fairytale (FeF), Fairytale exposed to Luhkas (FeL), and Fairytale exposed to Salome (FeS), with 16 replicates per treatment. In the Salome experiment, the treatments were: Salome exposed to Salome (SeS), Salome exposed to Luhkas (SeL), and Salome exposed to Fairytale (SeF), with 12 replicates per treatment. The treatments were randomly assigned and spatially arranged into 16 and 12 blocks, respectively, using a randomized block design to minimize environmental gradients and positional effects within the growing chamber.

### Plant sampling and processing

On the 25th day after planting, a time point at which volatile-mediated interactions between different cultivars typically produce the most pronounced differences in total dry weight (Ninkovic, 2003), plants were harvested for plant trait measurements and gene expression analyses. Plant samples were collected at the early tillering growth stage, corresponding to decimal code 20–22 of the Zadoks scale (Zadoks *et al.*, 1974). The plants were carefully removed by cutting the plastic sand tubes longitudinally and subsequently separated into two groups: roots and above-ground parts. Roots were gently washed with water to remove any adhering sand particles. The cleaned roots of each single plant were stored in a plastic bottle (100 ml) containing 10% alcohol in water solution, until size parameters were measured (max. 10 d). The detached leaves were carefully flattened using a book as a press. Leaves and roots were scanned using the image analysis system WinRHIZO Pro V 2007 (Regent Instruments, Québec, Canada). After scanning the leaves and roots, all plant samples (leaves, stems, and roots) were dried separately in an oven at 70 °C for 48 h and then spent 24 h at room temperature before they were weighed.

### Morphological parameters

To evaluate whether receiver plants alter their growth strategy in response to volatiles emitted by cultivars with different growth strategies, we measured the total dry biomass of both emitter and receiver plants (25 d after sowing; Zadoks growth stage 20–22, as described above). Specifically, we examined the biomass of slow-growing Fairytale and fast-growing Salome receivers exposed to volatiles from each of the three cultivars. A total of 15 plant traits were recorded or calculated, with particular focus on parameters related to plant development and growth. These include biomass production parameters, such as dry biomass for leaves, stems, and roots, each measured separately after drying to constant weight. The calculated values for above-ground biomass (sum of leaf and stem biomass) and total biomass (sum of leaf, stem and root biomass) were also included.

Additional growth parameters included mass fractions of leaf, root, and shoot, which were determined by dividing the dry mass of leaves, roots, or shoots by the total plant dry mass, respectively. The shoot-to-root ratio was calculated as the ratio of above-ground dry mass to root dry mass. Plant and stem heights were measured individually. Leaf surface area, representing the total surface area of all the leaves on a plant, was measured using the scanning and analysis software WinRhizo. Specific leaf area was derived as the ratio of leaf surface area to leaf dry mass (m^2^ kg^−1^). For root traits, total length and average root diameter were quantified using root scanning and analysis software WinRhizo. While the dry biomass measurements served as the primary focus of this study, these supplementary morphological attributes provided additional insights into the overall plant growth and development in response to the neighbor volatiles.

### RNA high-throughput library preparation and bioinformatic analysis

At the end of the 25 d experiment, 1 cm long samples were collected from the tips of the youngest leaf of each receiver plant and immediately placed in Eppendorf tubes and kept in liquid nitrogen. The youngest leaf was sampled because it provides a developmentally comparable tissue across plants (Skirycz *et al.*, 2010) and excised leaves have been reported to enhance detection of transcriptional changes (Brosset and Blande, 2022), making this tissue suitable for capturing VOC-induced transcriptional responses. For each treatment, leaf-tip samples from all receiver individuals were pooled into three composite samples. In the Fairytale-receiver experiment, 16 receiver plants per treatment were distributed across three composites; in the Salome-receiver experiment, 12 receiver plants per treatment were distributed across three composites. In total, this yielded 18 composite samples (6 treatments×3 composites) for RNA extraction and sequencing.

Total RNA was extracted using TRIzol reagent (Life Technologies) following the manufacturer’s instructions. mRNA for RNA sequencing was obtained by purification with the NEB mRNA isolation kit (New England Biolabs, Ipswich, MA, USA). Purified mRNA was used as input to prepare high-throughput RNA-seq libraries using the NEBNext Ultra II Directional RNA Library Prep Kit for Illumina (New England Biolabs). The resulting sequences were de-multiplexed, adapter trimmed, and quality controlled using TrimGalore (Krueger, 2015) (version 0.6.10) and FastQC (Andrews, 2010) (version 0.11.9). For gene expression analysis, RNA sequencing paired reads were aligned to the barley genome (Morex, version 3) using STAR (Dobin *et al.*, 2013) (version 2.7.11b), with default parameters. HTSeq-count was used to count reads per gene with the parameters -mode union -stranded no -minequal 10 and -nonunique none. Count tables obtained were used in DESeq2 (Love *et al.*, 2014) to infer significant expression with fit type set to parametric. All these tools were used through the Galaxy platform (Afgan *et al.*, 2018).

### Plant volatile sampling and analysis

For the headspace analysis, 12 pre-germinated seeds were transplanted into pots (9×9×7 cm) containing planting soil (P-jord, Örebro, Hasselfors Garden). To prevent unwanted volatile interactions between the three cultivars, each pot was placed in a transparent plastic cage (10×10×80 cm), from which air was pumped out of the chamber (see the description of the exposure system in the following). After 14 d of growth in the pots, reaching growth plant stage 12 of the Zadoks scale (Zadoks *et al.*, 1974), plant VOCs were sampled and analyzed. Volatiles were sampled at a comparable developmental stage under the same growing conditions for plant exposure, ensuring that the observed differences reflect qualitative variation in emission profiles rather than biomass-dependent variation. Headspace samples were collected in 10 replicates for each treatment. One Fairytale sample was lost due to technical issues, resulting in nine samples for that cultivar (*n*=9).

To compare the volatile profiles from barley cultivars used as emitters, the headspace from potted Salome, Luhkas, and Fairytale plants was sampled on adsorption tubes with a push–pull system for 24 h and analyzed by GC-MS. The upper part of each pot, containing 12 barley plants, was enclosed with a polyethylene terephthalate oven plastic bag (35×43 cm, Melita, Minden, Germany). Charcoal-filtered air was pushed into the oven bags with a flow of 600 ml min^−1^ while pulling the air out of the bags over a Tenax TA sample tube (80 mg, 60/80 mesh size, GLScience, Eindhoven, the Netherlands) at 400 ml min^−1^. Volatiles were released from the adsorbent tubes by thermal desorption with an Optics 3 Injector (GLScience) at 250 °C. The thermal desorbed compounds were separated using an Agilent 7890N GC system equipped with a HP-1MS capillary column (30×0.25 mm id×0.25 μm film thickness, 100% dimethylpolysiloxane) coupled to an Agilent 5975C mass spectrometer (Agilent Technologies, Inc., Santa Clara, CA, USA). Injection was employed using helium as carrier gas (Helium 6.0) with a flow of 1.3 ml min^−1^. The GC temperature program was as follows: initial oven temperature of 30 °C was held for 2 min, increased at a rate of 5 °C min^–1^ to 150 °C, followed by a rate of 10 °C min^–1^ to the final temperature of 250 °C, and held for 15 min. The GC inlet line temperature was 250 °C, and the ion source temperature was 180 °C. The quadrupole mass detector was operated in the electron impact (EI) mode at 70 eV, MS gain was set to 10. All data were obtained by collecting the full-scan mass spectra within the range of 40–500 *m/z*.

The volatile compounds from the chromatograms were identified and quantified with the ‘Automated Mass spectral Deconvolution and Identification System’ (AMDIS, V. 2.71; National Institute of Standards and Technology NIST, Boulder, CO, USA) following previously described methods (Gross *et al.*, 2019), in combination with the NIST MS Search program (Version 2.0) and the NIST/EPA/NIH Mass Spectral Library (NIST 08). Retention indices were calculated based on an *n*-alkane series (C8–C20). Identification criteria were applied as follows: match factor ≥80% and relative retention index deviation ≤5+0.01% from the reference value. The settings for deconvolution were component width, 32; adjacent peak subtraction, one; resolution, low; sensitivity, medium; shape requirements, low; level, very strong; maximum penalty, 25; and ‘no RI in library’, 20. Components with a signal to noise ratio <300 were excluded from the analysis. Because several detected compounds could not be chemically identified, we focused on relative peak areas rather than total emission rates; this approach reliably captures between-cultivar differences in VOC composition. Known background signals (e.g. siloxanes and other system-related contaminants) were identified based on characteristic fragment ions (e.g. *m/z* 73) and excluded from further analysis. No additional blank controls (pot with soil) were applied, as all compounds present in the exposure chambers were considered biologically relevant for the receiver plants.

### Statistical analysis

Statistical analyses and plotting of morphological attributes, such as total biomass production, were conducted using R (version 4.3.0) in the RStudio environment (version 2024.09.1+394, ‘Cranberry Hibiscus’). A two-way ANOVA was performed to identify significant differences among treatment groups at a 95% confidence level. The model included volatile treatment (i.e. identity of the neighboring plant cultivar) and block (i.e. experimental position within the growth chamber) as fixed factors. Interactions between treatment and block were initially tested but found to be non-significant, and were excluded from the final model. Block was included to control for spatial variation rather than to test for its direct effect. When a significant main effect of treatment was detected (*P*≤0.05), Tukey’s HSD post-hoc tests were used to assess pairwise differences among treatments.

Differential expression analysis of the epigenetic measurements was conducted using DESeq2 (version 1.44.0) in the R statistical environment (version 4.4.0). Significantly differentially expressed genes were identified based on appropriate statistical cut-offs, such as a false discovery rate (FDR) threshold of <0.05 and log2 fold change (log2FC) values < −1 or>1.

A random forest (RF) approach was used to compare the composition of plant VOCs released from the barley cultivars. *N*_tree_=10 000 bootstrap trees were drawn with *m*_yrt_=11 random variables at each node. A confusion matrix of the RF model and the corresponding classification errors were used to investigate the assignment accuracy of individual samples to the different cultivars. To identify the most important compounds responsible for the resolution of samples, the average mean decrease in accuracy (MDA) was calculated with the ‘importance’ function from the randomForest package (Liaw and Wiener, 2002).

The amount (peak area in the chromatogram) of the 10 most important VOCs that led to the separation of the volatile profiles from the different barley cultivars was compared using Kruskal–Wallis tests. Pairwise differences among cultivars were then assessed with Dunn’s test, which is appropriate for non-normally distributed data and provides a post-hoc procedure specifically designed for multiple pairwise comparisons following a significant Kruskal–Wallis result. Dunn’s test were performed using the ‘DunnTest’ function from the FSA package (Ogle *et al.*, 2015). *P*-values of multiple comparisons were adjusted with the Bonferroni method.

## Results

### Constitutive volatile organic compounds affect biomass production of divergent barley cultivars

To test whether constitutive VOCs modulate biomass production in neighboring plants, we conducted two independent exposure experiments using barley cultivars with contrasting growth rates: Fairytale (slow), Luhkas (intermediate), and Salome (fast). In each experiment, either Fairytale or Salome acted as the receiver.

**Fig. 2.**
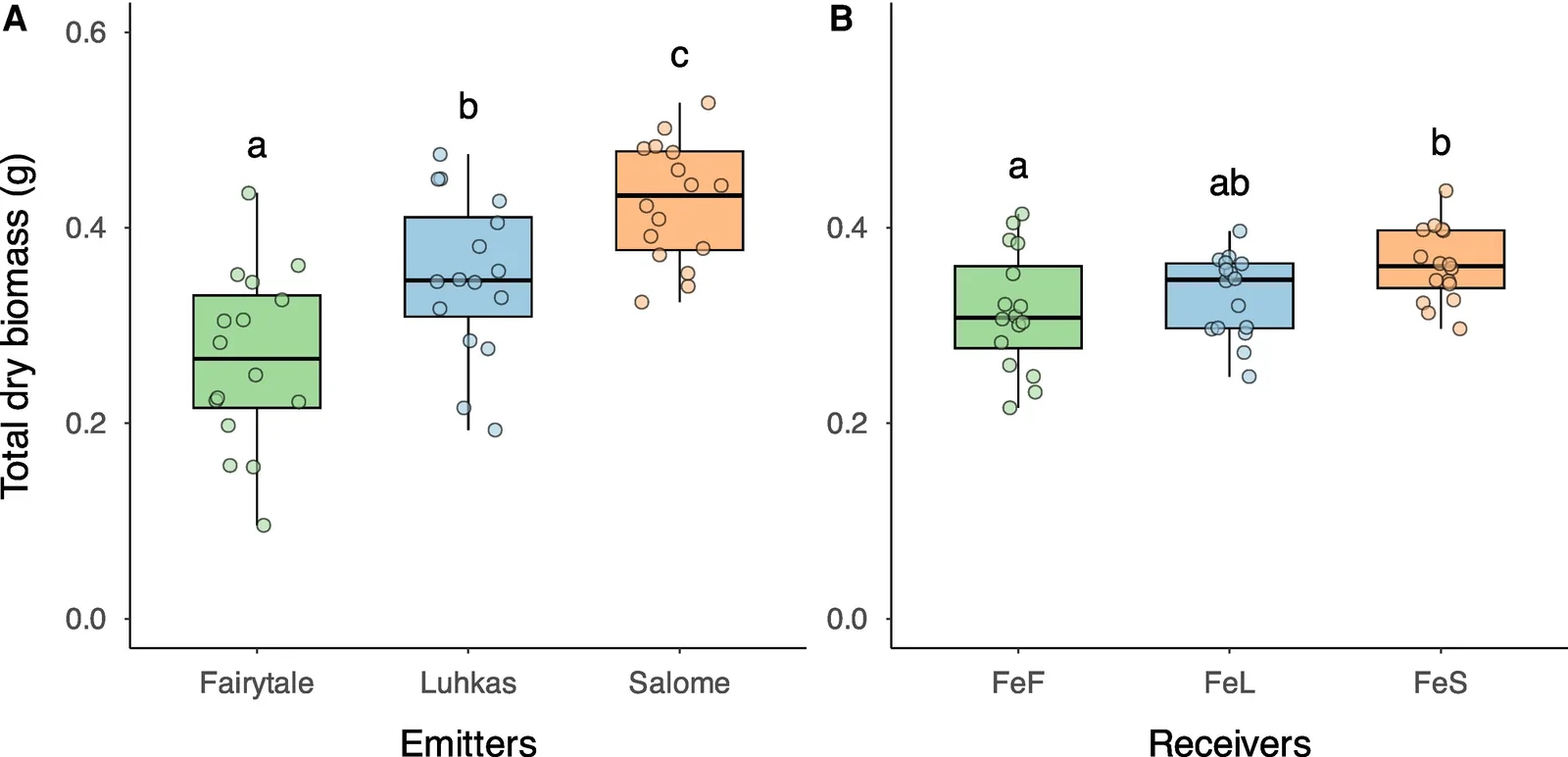
Biomass responses in emitters and Fairytale receiver plants. Total dry biomass of (A) emitter plants and (B) Fairytale receiver plants after exposure to volatiles from different cultivars: FeF=Fairytale exposed to Fairytale, FeL=Fairytale exposed to Luhkas, FeS=Fairytale exposed to Salome. Box plots show the interquartile range (IQR), median (horizontal line), and whiskers extending to 1.5× the IQR. Each point represents one plant (*n*=16). Different letters indicate statistically significant differences (*P*≤0.05, two-way ANOVA with Tukey’s HSD post-hoc test).

In the first experiment, the slow-growing Fairytale was exposed to the constitutive VOCs emitted by Fairytale (FeF), Luhkas (FeL), or Salome (FeS). The biomass of emitters differed significantly among cultivars in this experiment [Fig. 2A, ANOVA: *F* (2, 30)=20.42, *P*>0.001], with Fairytale producing less biomass than Luhkas (*P*=0.006) and Salome (*P*<0.001), and Luhkas producing less than Salome (*P*=0.014). Receiver biomass (Fig. 2B) was influenced by emitter identity [ANOVA: *F* (2, 30)=3.97, *P*=0.029]. Fairytale plants exposed to constitutive VOCs from Salome had significantly higher biomass than those exposed to VOCs from Fairytale (*P*=0.025), while exposure to Luhkas did not differ significantly from the other treatments (*P*>0.05).

**Fig. 3.**
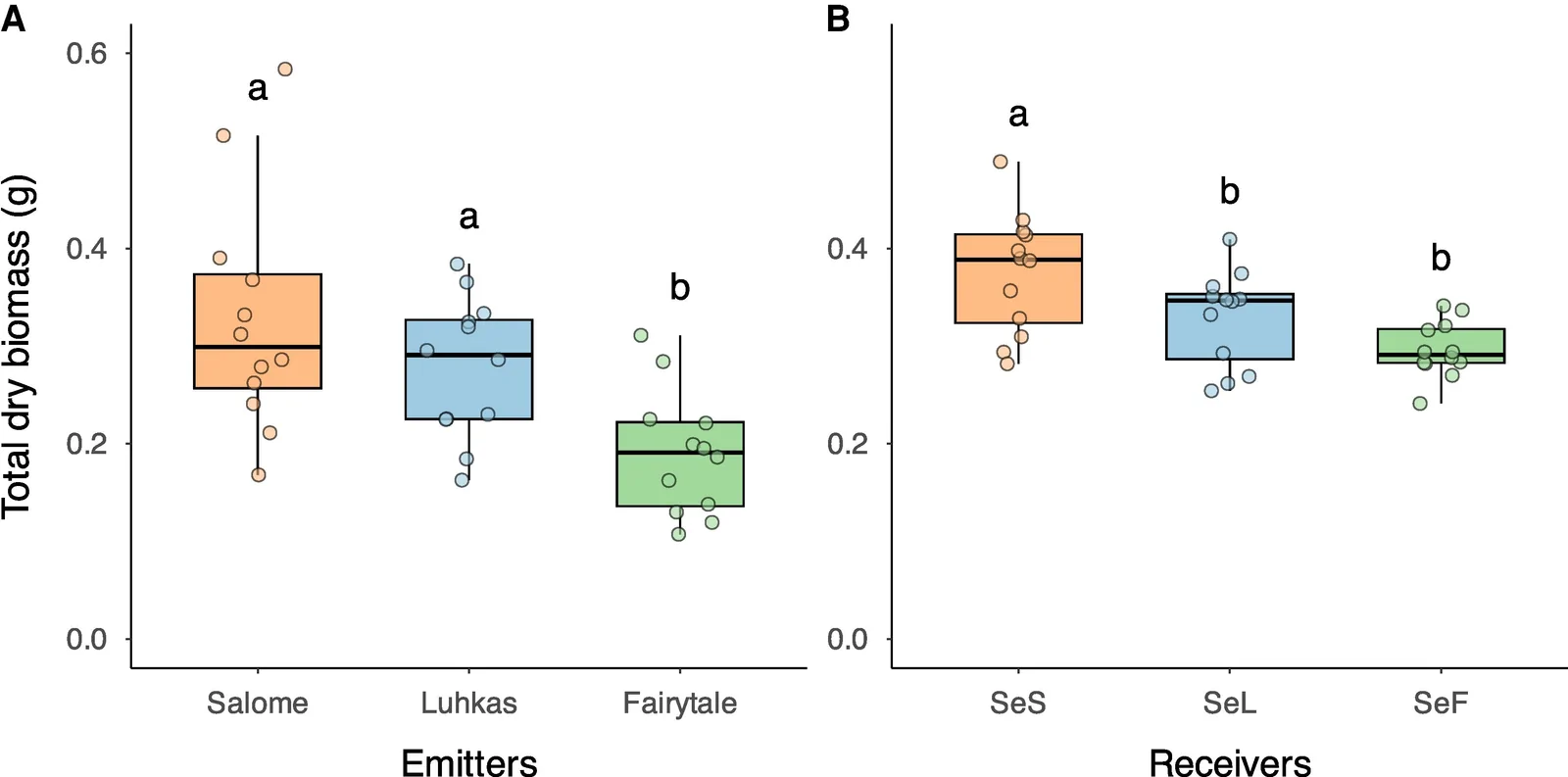
Biomass responses in emitter plants and Salome receiver plants. Total dry biomass of (A) emitter plants and (B) Salome receiver plants after exposure to volatiles from different cultivars: SeS= Salome exposed to Salome, SeL=Salome exposed to Luhkas, SeF=Salome exposed to Fairytale. Box plots show the interquartile range (IQR), median (horizontal line), and whiskers extending to 1.5× the IQR. Each point represents one plant (*n*=12). Different letters indicate statistically significant differences (*P*≤0.05, two-way ANOVA with Tukey’s HSD post-hoc test).

In the second exposure experiment, the fast-growing cultivar Salome was exposed to constitutive VOCs emitted by Salome (SeS), Luhkas (SeL), or Fairytale (SeF). The biomass of emitters was significantly different between cultivars [Fig. 3A, ANOVA: *F* (2, 22)=9.48, *P*=0.001]. Fairytale emitters produced less biomass than Luhkas (*P*=0.032) and Salome (*P*<0.001), while Luhkas and Salome did not differ significantly (*P*>0.05). The biomass of receiving Salome plants was significantly influenced by emitter identity [Fig. 3B, ANOVA: *F* (2, 22)=9.74, *P*<0.001]. Salome plants exposed to constitutive VOCs from Fairytale had lower biomass than those exposed to Salome (*P*<0.001). Exposure to Luhkas also reduced biomass relative to Salome (*P*=0.046). No significant difference was found between exposure to Fairytale or Luhkas (*P*>0.05). Leaf, stem, and root biomass followed the same pattern as total biomass, indicating no detectable shift in allocation among plant parts (Supplementary Tables S1, S2).

### Additional morphological traits show limited response to volatile organic compound exposure

Beyond total biomass, we assessed a range of morphological and allocation traits to determine whether exposure to neighbor VOCs affected other aspects of plant development. In the experiment where Fairytale acted as the receiver, no significant differences were observed among treatments for leaf, shoot, or root mass fractions (Supplementary Table S3). The shoot-to-root ratio was significantly higher in Salome emitters compared with Fairytale (*P*=0.034) and Luhkas (*P*=0.036). Leaf surface area increased significantly across emitter cultivars from Fairytale to Luhkas to Salome, with all pairwise comparisons being significantly different (F–L=0.008, F–S<0.001, L–S<0.001). Specific leaf area, plant height, stem heights, and average root diameter did not vary significantly across treatments. Root length increased significantly across emitter cultivars (F–L=0.017, F–S<0.001, L–S=0.042). Overall, emitter identity did not show any significant treatment effects in Fairytale receivers for these additional traits.

In the Salome-receiver experiment, leaf and shoot mass fractions of the emitters were significantly lower in Luhkas than in Salome (leaf, *P*=0.034; shoot, *P*=0.002), with Fairytale showing intermediate values (Supplementary Table S4). Receiver plants showed no significant differences in leaf or shoot mass fractions. Root mass fraction and shoot-to-root ratio exhibited no significant treatment effects. Leaf surface area in emitters increased significantly from Fairytale to Luhkas to Salome (F–L=0.04, F–S<0.001, L–S=0.015). Among receivers, SeF had a lower leaf surface area than SeS (*P*=0.004), with SeL being intermediate. Specific leaf area was largely consistent across treatments, apart from a small but significant difference between Luhkas and Salome emitters (*P*=0.045). For plant height, SeS was significantly taller than both SeL (*P*=0.003) and SeF (*P*=0.023), with no significant difference between SeF and SeL. For stem height, SeL was significantly shorter than both SeS (*P*<0.001) and SeF (*P*=0.047), whereas SeS and SeF did not differ significantly. Root length in emitter plants was significantly lower in Fairytale compared with Salome (*P*=0.002) and Luhkas (*P*=0.011). Among receivers, SeS had significantly greater root length than both SeF (*P*=0.003) and SeL (*P*=0.015). Average root diameter showed no treatment effects. Overall, emitter identity altered only a subset of traits in Salome receivers, specifically leaf surface area, plant height, stem height, and root length, while all other measured parameters showed no significant or consistent treatment-related effects.

### Transcriptional response to constitutive volatile organic compound exposure mimics biomass production

**Fig. 4.**
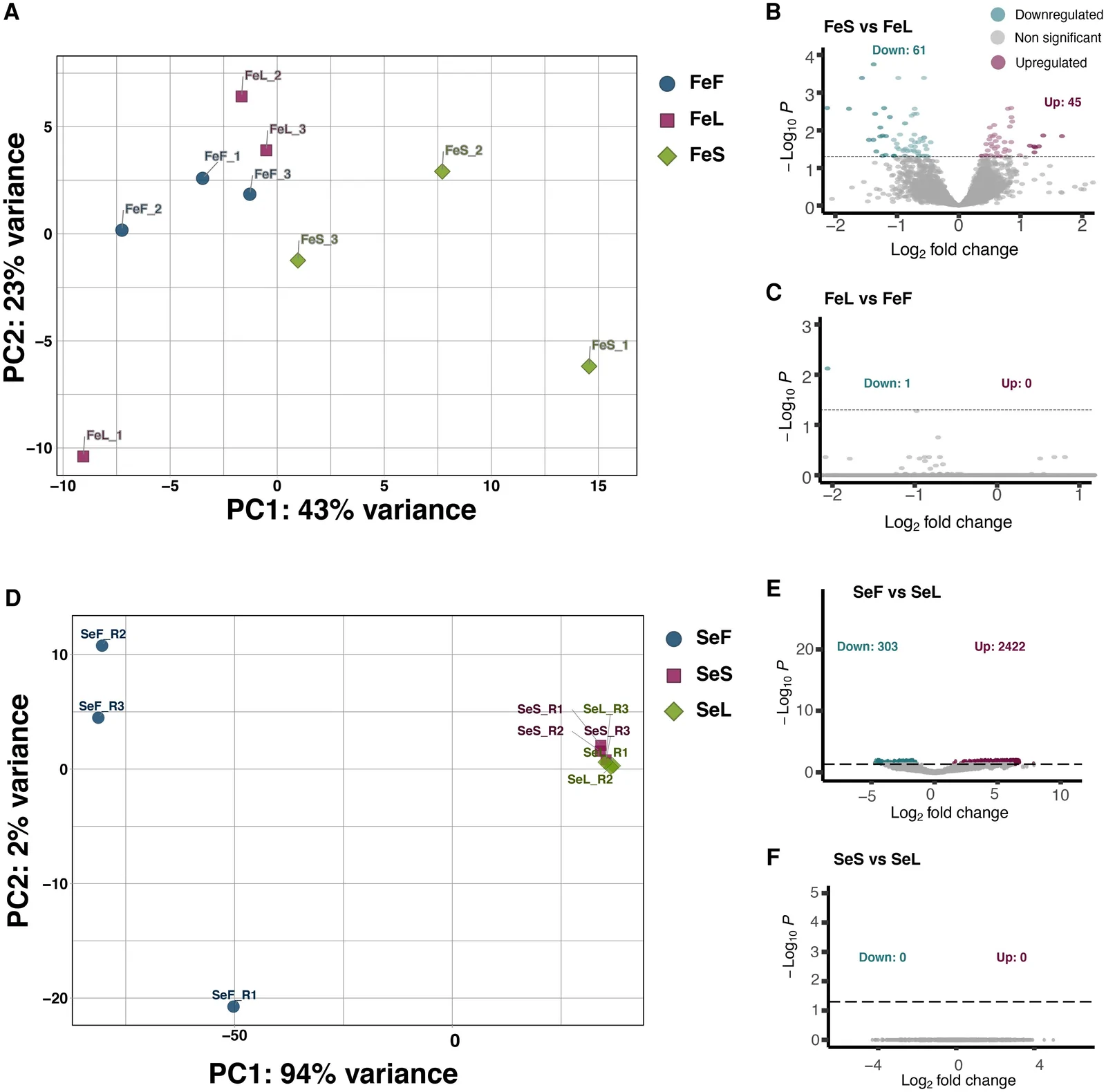
Principal component analysis of RNA sequencing samples from Fairytale and Salome receiver combinations and volcano plots showing differential gene expression. (A) Principal component analysis (PCA) of RNA libraries generated from Fairytale receiver combinations. Numbers represent bioreplicates analyzed for each combination. (B) Volcano plots showing the up- and down-regulated genes in FeS compared with FeL and in (C) FeL compared with FeF. (D) PCA for the RNA libraries produced from Salome receiver combinations. Numbers indicate bioreplicates analyzed for each combination. (E) Volcano plots showing the up- and down-regulated genes in SeF compared withSeL, and in (F) SeS compared with SeL.

**Fig. 5.**
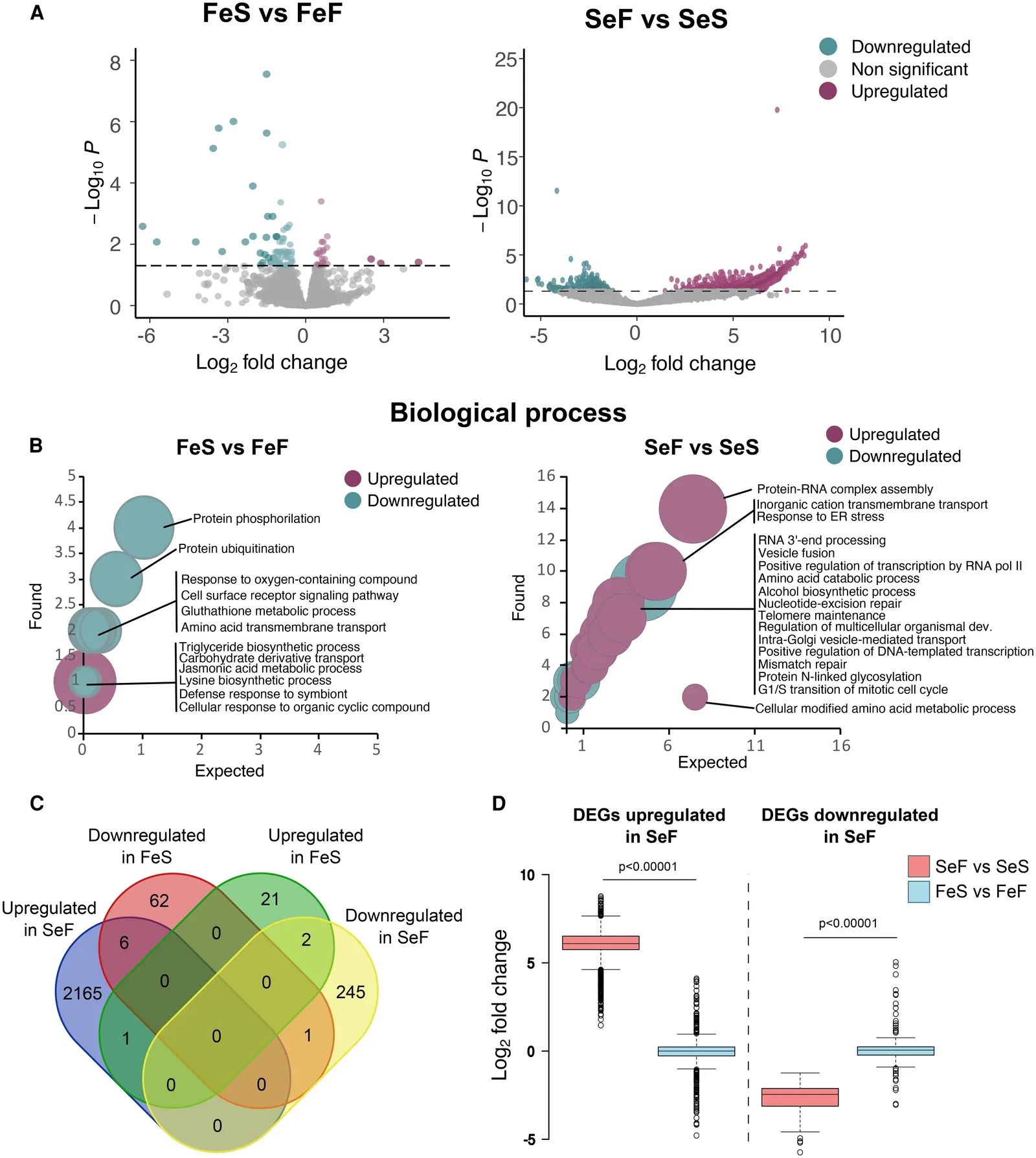
Gene expression responses to VOC exposure. (A) Volcano plots showing the up- and down-regulated genes in both experiments. Left panel: FeS compared with FeF. Right panel: SeF compared with SeS. (B) Gene Ontology functional enrichment plots, showing categorization of biological processes in the comparisons between FeS and FeF (left panel), and SeF and SeS (right panel). (C) Venn diagram showing the overlap between genes up- and down-regulated in the SeF and FeS. (D) Expression level of DEGs up- and down-regulated in SeF, in both the comparisons SeF versus SeS and FeS versus FeF. The *P*-value was calculated through an unpaired, unequal variance *t*-test.

The level of physiological changes observed in our experimental setup prompted us to explore the genome-wide transcriptional reprogramming taking place in receiver plants exposed to constitutive VOCs. To that aim we prepared and sequenced high-throughput mRNA libraries from the two exposure experiments with a focus on receiver plants. A principal component analysis (PCA) of the RNA libraries (Fig. 4A, D) was carried out and, as expected from this PCA, differential expression analysis (adjusted *P*-value <0.05) revealed significant differences in gene expression between treatments (Fig. 5A).

Fairytale exposed to Salome (FeS) exhibited a higher number of down-regulated genes (24 up-regulated and 69 down-regulated genes compared with its control Fairytale exposed to Fairytale, FeF). Similarly, when FeS was compared with FeL, we observed 45 up-regulated and 61 down-regulated genes (Fig. 4B), indicating that FeS receiver plants show the highest transcriptional changes from all the Fairytale receiver combinations. As expected, FeL compared with FeF showed only one up-regulated gene (Fig. 4C). Interestingly, Salome exposed to Fairytale (SeF) exhibited the opposite transcriptional response, with a higher number of up-regulated genes (2172 up-regulated genes and 248 down-regulated genes compared with Salome exposed to Salome, SeS). This result was consistent when SeF receivers were compared with SeL, showing a similar tendency to gene up-regulation (2422 up-regulated genes and 303 down-regulated genes) (Fig. 4E). Similar to the result observed in Fairytale used as receiver, the comparison between SeS and SeL revealed no significant differences at the transcriptional level (Fig. 4F).

Gene Ontology (GO) functional enrichment analyses (Fig. 5B) were conducted to provide a broad overview of the functional roles of the up- and down-regulated genes in the plants. In Fairytale exposed to Salome compared with Fairytale exposed to Fairytale (FeS versus FeF), the primary functional categories (according to the biological process categorization) associated with the down-regulated genes were involved in protein homeostasis (protein phosphorylation, ubiquitination, amino acid transmembrane transport, triglyceride biosynthetic process, etc.) and the response to stress (response to oxygen-containing compound, defense response to symbiont, cellular response to organic cyclic compound, etc.). In comparison with Salome as the receiving cultivar, genes differentially expressed were associated with processes involved in RNA homeostasis (protein–RNA complex assembly, RNA 3'-end processing, regulation of transcription, etc.), cellular transport (inorganic cation transmembrane transport, vesicle fusion, intra-Golgi vesicle-mediated transport, etc.), DNA replication (telomere maintenance, mismatch repair, and G_1_/S transition of the mitotic cell cycle), and protein homeostasis (amino acid catabolic process).

To further compare the transcriptional responses in the two receiver cultivars, we explored the overlap between up- and down-regulated genes on each comparison (Fig. 5C). This showed that there was a small overlap of differentially expressed genes (DEGs) up-regulated in SeF that were also down-regulated in the opposite situation FeS (six genes, 8.7% of the total down-regulated DEGs in FeS). Similarly, a number of down-regulated genes in SeF were up-regulated in FeS (two genes, 8.3% of the total up-regulated DEGs in FeS). Next, we explored the changes in total expression for SeF up- and down-regulated genes (Fig. 5D). Interestingly, we found that up-regulated DEGs in SeF have significantly lower expression levels in FeS, while down-regulated DEGs in FeS showed significantly higher expression levels than in SeF (Fig. 5D). In summary, our transcriptomic analysis indicated that SeF and FeS experienced antagonistic transcriptional changes and a significant increase in the expression of genes associated with categories that explain their physiological changes after exposure to specific emitters.

### Functional annotation of overlapping differentially expressed genes

**Fig. 6.**
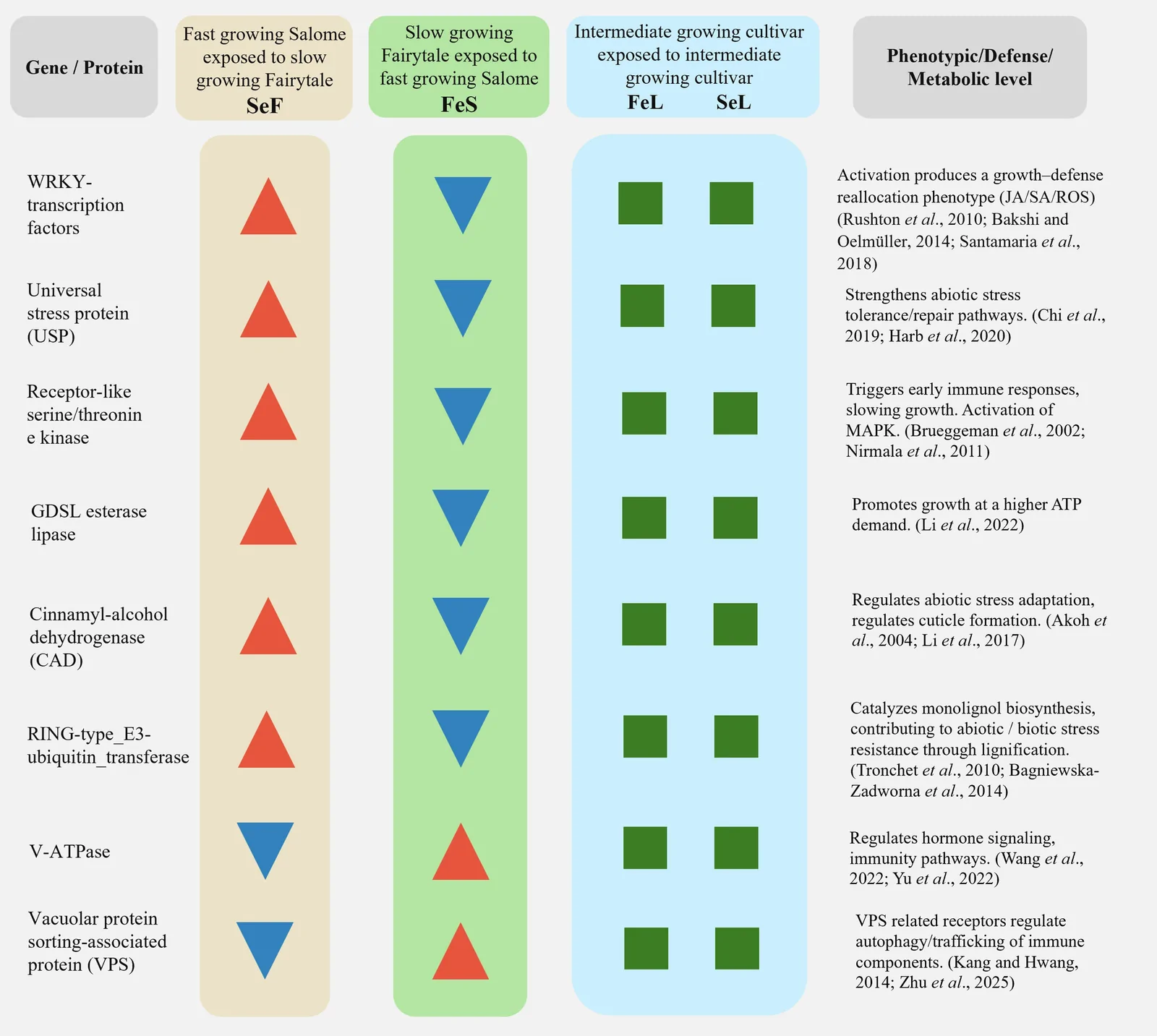
Growth- and defense-associated gene expression in barley cultivars with contrasting growth strategies. Selected overlapping differentially expressed genes (DEGs) across cultivars with contrasting growth strategies. Upward triangles indicate significant up-regulation, downward triangles significant down-regulation, and squares no significant change in gene expression following exposure. When the fast-growing Salome was exposed to the slow-growing Fairytale (SeF), gene expression patterns indicated a shift in resource allocation from growth toward defense. The reciprocal exposure (Fairytale exposed to Salome, FeS) showed the opposite pattern, with expression favoring growth-related processes. In contrast, exposures between cultivars of similar growth rates, Fairytale exposed to Lukhas (FeL) and Salome exposed to Lukhas (SeL), resulted in no significant changes in gene expression.

To further explore the biological significance of the transcriptional responses, we examined the putative functions of genes differentially expressed in both receiver cultivars (Fig. 6). When receiver plants were exposed to cultivars with markedly contrasting growth strategies, the regulation of growth- and defense-associated pathways was particularly pronounced. Strikingly, exposures between cultivars of similar growth rates, Fairytale exposed to Luhkas (FeL) and Salome exposed to Luhkas (SeL), resulted in no significant changes in expression for any of these genes.

Among the genes differentially expressed between SeF and FeS, two functional clusters show opposing patterns (Fig. 6). Genes up-regulated in SeF and down-regulated in FeS include those encoding proteins with roles in stress signaling and defense: WRKY transcription factors, whose activation is associated with a growth–defense reallocation phenotype (Rushton *et al*., 2010; Bakshi and Oelmüller, 2014; Santamaría *et al*., 2018), a universal stress protein (USP) (Chi *et al*., 2019; Harb *et al*., 2020), a receptor-like serine/threonine kinase (Brueggeman *et al*., 2002; Nirmala *et al*., 2011), a GDSL esterase lipase (Li *et al*., 2022), a cinnamyl alcohol dehydrogenase (CAD) (Akoh *et al*., 2004; Li *et al*., 2017), and a RING-type E3 ubiquitin ligase (Tronchet *et al*., 2010; Bagniewska-Zadworna *et al*., 2014). In contrast, genes up-regulated in FeS and down-regulated in SeF include those encoding a V-ATPase, associated with regulation of hormone signaling and immunity pathways (Wang *et al*., 2022; Yu *et al*., 2022), and a vacuolar protein sorting-associated protein (VPS), associated with regulation of autophagy (Kang and Hwang, 2014; Zhu *et al*., 2025). It should be noted that the functional roles attributed to these genes are largely inferred from studies of homologs in other plant species, and their specific functions in barley remain to be confirmed experimentally. The antagonistic regulation of these genes nonetheless provides molecular support for the opposing shifts in biomass production observed between the two receiver cultivars, and suggests that these phenotypic differences reflect broader reallocations between growth- and defense-associated processes.

### Constitutive volatile organic compound profiles differ between barley cultivars with divergent growth strategies

**Fig. 7.**
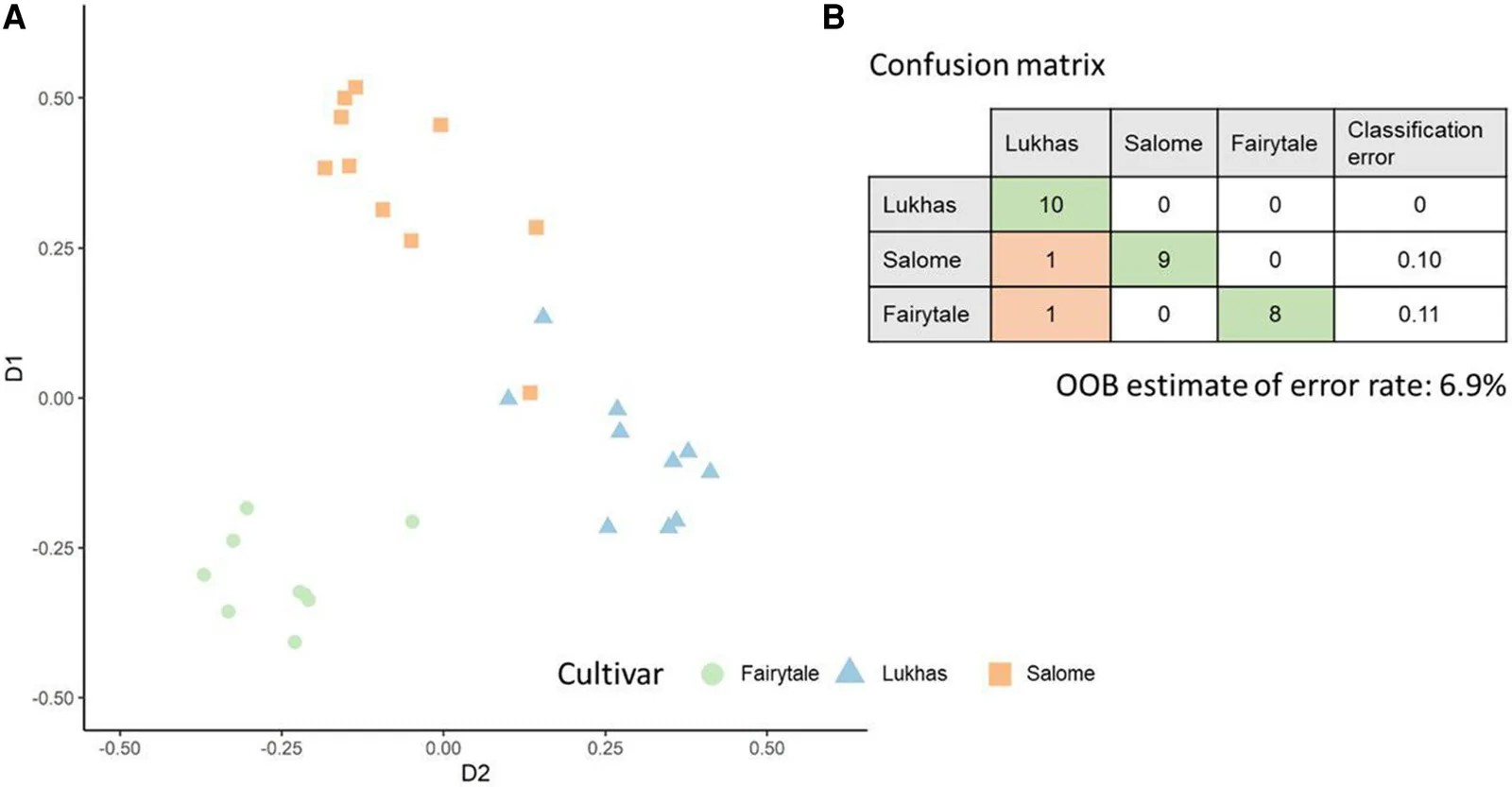
Classification of cultivar-specific VOC profiles. (A) Multi-dimensional scaling (MDS) plot of a random forest (RF) model using the proportion of VOCs released from barley cultivars Fairytale (circles, *n*=9), Luhkas (triangles, *n*=10), and Salome (squares, *n*=10). (B) Corresponding confusion matrix of sample classification of the RF model. The matrix shows the correct and false assignment of cultivar VOC profiles and the classification error for each cultivar.

VOC composition varies both across plant species (Vivaldo *et al.*, 2017) and among cultivars within species (Dahlin *et al.*, 2018). To examine the differences among the three barley cultivars in this study, we analyzed the headspace composition of 115 annotated volatile compounds using GC-MS, yielding 94, 100, and 91 annotated VOCs for Fairytale, Luhkas, and Salome, respectively (Supplementary Table S5). An RF model achieved 93.1% accuracy in classifying volatile profiles from undamaged plants. Constitutive VOC profiles were clearly separable among Salome, Fairytale, and Luhkas (Fig. 7A), with Salome and Luhkas clustering more closely together than they did with Fairytale. All Luhkas samples were assigned correctly with no confusion with other cultivars (Fig. 7B). Salome VOC profiles were classified with 90% accuracy; a single sample was misclassified as Luhkas (Fig. 7B). Eight out of nine volatile samples from Fairytale were correctly classified as Fairytale, while one was wrongly assigned as Luhkas (Fig. 7B). Notably, VOC profiles from Salome and Fairytale were consistently distinguishable, showing the greatest separation in both clustering and classification (Figs. 7A, B).

The 10 VOCs which had the greatest influence on the separation of the cultivars (MDA value) were emitted in significantly different quantities from the three cultivars [Kruskal–Wallis, df=2, retention index (RI) 1160: χ^2^=18.26, *P*<0.001; benzyl nitrile: χ^2^=18.99, *P*<0.001; 1-octen-3-ol: χ^2^=24.06, *P*<0.001; benzothiazole: χ^2^=6.36, *P*=0.042; tetradecane: χ^2^=8.12, *P*=0.017; octanal: χ^2^=15.52, *P*<0.001; dodecane: χ^2^=8.43, *P*=0.015; linalool: χ^2^=13.81, *P*=0.001; RI 955: χ^2^=13.45, *P*=0.001; nonanal: χ^2^=14.62, *P*<0.001].

**Fig. 8.**
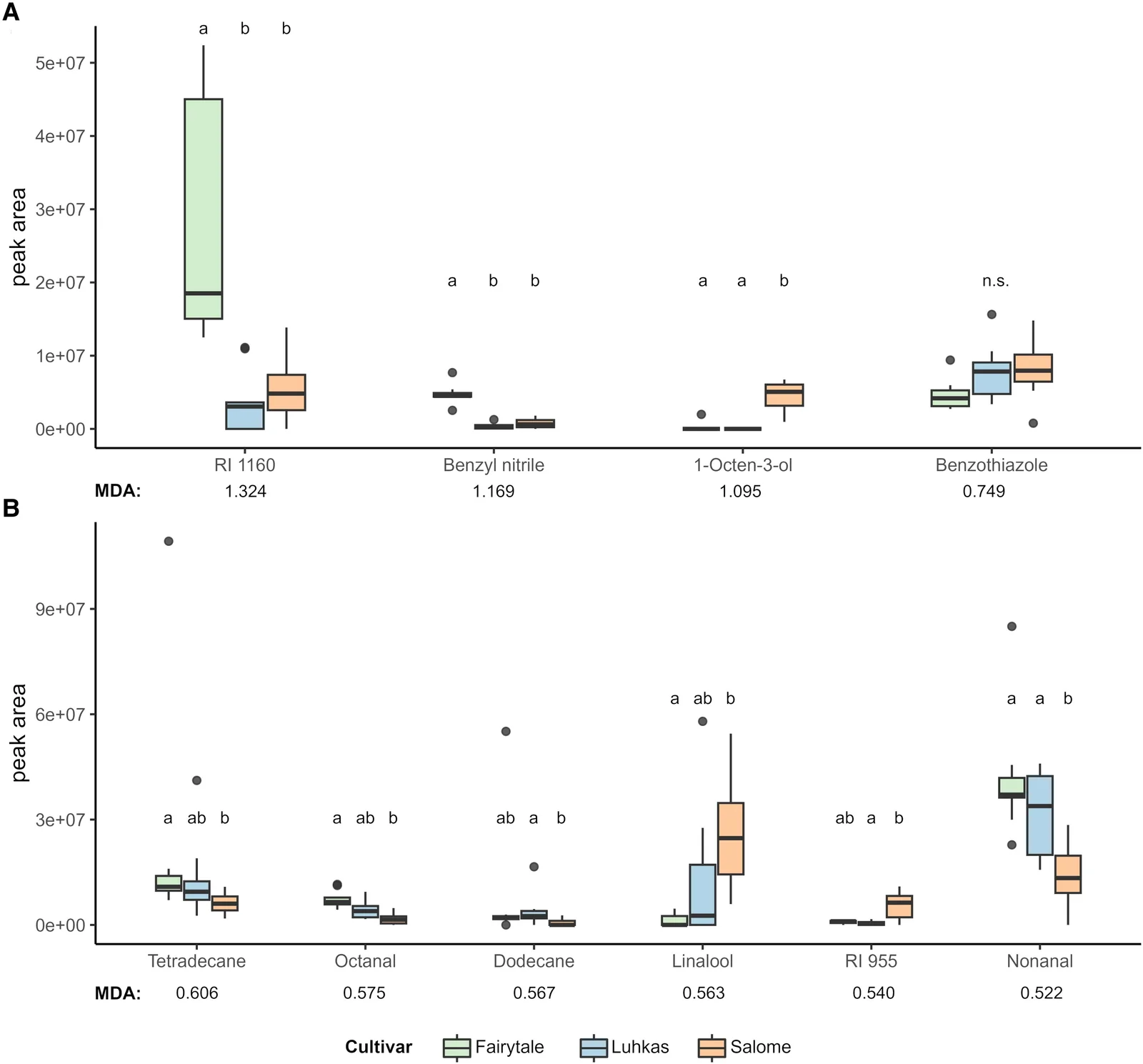
The most influential VOCs in cultivar classification. Peak area (amount) of VOCs released from three different barley cultivars: Fairytale (*n*=9), Luhkas (*n*=10), and Salome (*n*=10), which have the greatest influence on the separation of cultivars by the random forest model. The impact on the separation is evaluated by the mean decrease in accuracy (MDA). Boxes correspond to the 25th and 75th percentiles, medians are shown as lines, and whiskers extend to 1.5 times the interquartile ranges. Dots represent outliers. Letters indicate significant differences (*P*<0.05) of pairwise comparisons with Dunns’ test with Bonferroni correction as the post-hoc test after signficant Kruskal–Wallis rank sum test.

Benzyl nitrile and the unidentified compound RI 1160 were released significantly more from Fairytale compared with Luhkas and Salome (Fig. 8A). The release of 1-octen-3-ol was characteristic for Salome plants (Fig. 8A). Tetradecane, octanal, and linalool were released in different amounts from Fairytale compared with Salome, while there was no significant difference from both cultivars compared with Luhkas (Fig. 8B). In chromatograms of samples from Fairytale and Luhkas, significantly greater peak areas (amounts) of nonanal were detected compared with samples from Salome (Fig. 8B). In conclusion, each cultivar produced a distinct constitutive VOC profile, with key compounds varying in both identity and abundance. These differences are likely to underpin the cultivar-specific growth and transcriptional responses observed in receiver plants.

## Discussion

Our findings provide a proof-of-concept that constitutive VOCs can function as reliable informational cues, conveying both the identity and competitive potential of the emitter even in the absence of external stress. The asymmetric responses observed in receiver plants, depending on the identity of the emitter, elicited marked shifts in biomass production and gene expression patterns. Because changes in total biomass were consistent across leaves, stems, and roots, receivers appear to adjust their overall growth strategy rather than reallocating biomass or converging toward the morphology of the emitter. This indicates that plants may exploit VOC-mediated signaling to detect potential competitors and to proactively adjust their own growth and defense strategies. These results support the view that VOC perception represents a dynamic assessment process, enabling plants to anticipate competitive interactions and to modulate internal resource allocation in advance.

Undamaged plants emit distinct VOC profiles reflecting their inherent growth strategies (Ninkovic *et al.*, 2016; Dahlin *et al.*, 2018). Our findings demonstrate that neighboring plants can detect these constitutive VOC cues and adjust their growth–defense allocation in response. This aligns with the ecological principle to balance resources between growth and defense (Herms and Mattson, 1992; Züst and Agrawal, 2017). In our experiments, the slow-growing cultivar Fairytale increased biomass when exposed to the fast-growing Salome (FeS), whereas Salome showed reduced biomass when exposed to Fairytale (SeF) (Figs 2, 3). The intermediate-growing Luhkas consistently showed moderate responses. This pattern supports the context dependence of plant responses to neighbor VOCs, a concept reflected in studies reporting both growth stimulation and inhibition following exposure to HIPVs (Cofer *et al.*, 2018; Freundlich *et al.*, 2021). The magnitude and direction of the observed biomass changes were closely associated with the differences in growth strategies between emitter and receiver cultivars, underscoring the ability of plants to decode subtle chemical cues to fine-tune allocation accordingly. This trend was reinforced by transcriptomic analyses, which revealed more DEGs detected in interactions between Fairytale and Salome compared with those involving Luhkas (Fig. 4). These DEGs were largely linked to cell growth and defense activation, mirroring previous observations that up-regulation of resistance genes can compromise growth (Cipollini and Heil, 2010).

These findings reflect adaptive resource allocation strategies consistent with growth–defense trade-offs (Herms and Mattson, 1992; Züst and Agrawal, 2017). Fast-growing plants typically invest in rapid biomass accumulation over defense, while slow-growing plants allocate more to defense (Herms and Mattson, 1992). Therefore, exposure to a slow-growing neighbor may convey lower competitive pressure and prompt defense investment, while exposure to a fast-growing neighbor may elicit a growth-oriented response, potentially at the cost of inducible resistance (Fig. 6). Similar effects have been reported for induced VOCs released by damaged plants, such as in *Artemisia tridentata*, that increased pest resistance but reduces growth in response to neighbor volatiles (Karban, 2017), or specific HIPVs such as (*Z*)-3-hexenyl acetate that mediate growth–defense trade-offs (Engelberth and Engelberth, 2019; Freundlich *et al.*, 2021). However, unlike these stress-induced signals, our findings demonstrate that such physiological shifts can also be triggered by constitutive VOCs emitted by undamaged plants. While our proof-of-concept design focused on three barley cultivars, evaluating a broader range of genotypes would be a valuable next step for assessing the generality of these responses.

The three barley cultivars examined in our study emitted distinct constitutive VOC profiles (Fig. 7), with the most pronounced chemical divergence observed between fast-growing Salome and slow-growing Fairytale. These cultivars were selected for their contrasting growth strategies, and our multivariate analyses confirm that these phenotypic differences are linked to distinct, cultivar-specific VOC profiles. This chemical dissimilarity probably drives the differential responses in receiver plants, contributing to the modulation in growth–defense trade-offs. Notably, benzyl nitrile and a chemically unidentified compound (RI 1160) were characteristic components of the VOC profile of Fairytale (Fig. 8). Benzyl nitrile is a well-documented HIPV known for its insect-repellent properties (Dong *et al.*, 2011; Twidle *et al.*, 2022; Qian *et al.*, 2023), and its constitutive emission by Fairytale may reflect a defense-oriented metabolic strategy. Although its role in VOC-mediated interactions among undamaged plants has not yet been investigated, our findings suggest that it may function as a cue influencing growth–defense allocation in neighboring plants. Tetradecane and octanal also emerged as potentially significant compounds of interest, as their emission levels varied markedly across cultivars. These differences are consistent with the notion that a minimum specific VOC concentration is required to surpass threshold levels to activate chemosensory perception and physiological responses in neighboring plants (Arimura and Uemura, 2025).

Testing the biological activity of individual compounds and defined synthetic mixtures, including their concentration-dependent effects, will be essential to establish causal relationships between specific VOCs and the observed plant responses. Furthermore, it should be considered that the applied analytical approach is limited to compounds detectable with the selected method. Small and highly volatile signaling molecules such as ethylene are not captured and therefore cannot be excluded as contributing factors. Future studies should therefore include known signaling components such as ethylene or other phytohormones in synthetic mixtures to investigate their combined effects on plant responses.

The observed changes in receiver gene expression and growth patterns occurred in the absence of physical damage or biotic stress, indicating that constitutive VOCs alone can activate pathways typically associated with induced defense (Fig. 1). Given that defense pathways can be induced in undamaged receivers, and considering the reciprocal trade-off between growth and defense, we speculate that such induced responses may significantly impact competitive dynamics. Competition is among major stressors in plants (Goldberg and Barton, 1992), and VOC-mediated perception of neighbor vigor could provide a low-cost mechanism for early adjustments of physiological strategies. From an agricultural perspective, these findings suggest that the selection of cultivar mixtures could be guided by their contrasting growth strategies and VOC profiles, potentially optimizing both yield and pest resistance without reliance on external inputs. In this context, constitutive VOCs may prime the growth or defense strategy of the receiver plants based on the perceived competitive potential of its neighbors.

Gene expression analyses showed that plants actively reprogrammed their transcriptomes in response to constitutive VOCs emitted by undamaged neighbors, with patterns of gene regulation varying across emitter–receiver combinations. When Fairytale was exposed to VOCs from Salome (FeS), the majority of DEGs were down-regulated, particularly those involved in protein maintenance, stress responses, and phosphorylation, suggesting reduction in defense investment. In contrast, Salome exposed to Fairytale VOCs (SeF) showed broad up-regulation of DEGs associated with RNA processing, DNA replication, and protein metabolism, potentially reflecting enhanced developmental activity or increased readiness for stress. Notably, Salome up-regulated multiple WRKY transcription factors in response to Fairytale VOCs, known to mediate plant resistance to aphids (Wani *et al.*, 2021). These results are in line with previous findings that VOC exposure can reduce aphid performance (Dahlin *et al.*, 2018; Kheam *et al.*, 2023). Further, a universal stress protein (Chi *et al.*, 2019) and a receptor-like serine/threonine kinase (Brueggeman *et al.*, 2002, 2006; Nirmala *et al.*, 2006), key players in biotic stress response, were up-regulated in SeF but down-regulated in the opposite direction. Other genes such as a WAK family protein (Tripathi *et al.*, 2021) and an AB hydrolase-1 domain protein (Zakhrabekova *et al.*, 2023) may influence broader development and stress responses. In addition, a RING-type E3 ubiquitin ligase (You *et al.*, 2016) may contribute to disease resistance and the coordination of growth–defense trade-offs. Interestingly, two genes up-regulated in FeS, namely a predicted protein and a V-ATPase involved in turgor regulation and growth (Li *et al.*, 2022), were down-regulated in SeF. These contrasting patterns underscore cultivar-specific responses and support the hypothesis that VOC perception shapes physiological priorities in growth and defense.

Our results can be interpreted within both the trade-off hypothesis and the simple differential signal transduction hypothesis. The observed biomass changes and reciprocal transcriptional shifts between Fairytale and Salome support the trade-off model, whereby increased defense investment entails a reduction in growth, and vice versa. Simultaneously, the cultivar-specific transcriptional reprogramming, including opposite regulation of stress-related genes in reciprocal exposures, aligns with the simple differential signal transduction hypothesis, suggesting that VOC perception modulates shared signaling pathways in different directions depending on emitter identity. Together, these frameworks demonstrate that constitutive VOCs can act both as anticipatory cues of competition and as modulators of internal signaling trade-offs, thereby shaping adaptive plant–plant interactions.

The ability to perceive and respond to neighbor VOCs is essential for optimizing plant fitness through rapid and context-dependent physiological adjustments (Ninkovic *et al.*, 2021). Our findings further support the hypothesis that growth–defense trade-offs are not static properties of individual plants but are context dependent and dynamically regulated in response to VOC cues from neighboring plants. Receiver plants in our system appear to assess the competitive capacity of their neighbors through their constitutive VOC profile and adjust their growth strategies accordingly. Exposure to VOCs from fast-growing, highly competitive emitters promotes investment in growth at the expense of defense, whereas exposure to VOCs from slow-growing, low-competition emitters results in reduced growth and increased investment in defense. These context-dependent strategic adjustments resemble those observed in response to herbivory (Engelberth *et al.*, 2004), pathogen presence (Hammerbacher *et al.*, 2019), or abiotic stress (Caparrotta *et al.*, 2018; Markovic *et al.*, 2019), all of which can modulate VOC production and prime neighboring plants for future challenges. Collectively, our findings demonstrate that even VOCs constitutively released by undamaged plants can serve as ecologically meaningful signals, modulating the growth and defense priorities of conspecific neighbors and shaping competitive dynamics within plant communities.

## Supplementary Material

erag252_Supplementary_Data

## Data Availability

The raw primary data supporting this study were not made publicly available at the time of publication; however, these data will be made available by the corresponding author Velemir Ninkovic, without undue reservation.

## Supplementary data

The following supplementary data are available at *JXB* online.

Fig. S1. Twin-chamber volatile exposure system.

Table S1. Leaf, stem, and root biomass in Fairytale exposed to volatiles from different emitter cultivars (Fairytale, Luhkas, or Salome).

Table S2. Leaf, stem, and root biomass in Salome exposed to volatiles from different emitter cultivars (Salome, Luhkas, or Fairytale).

Table S3. Overview of measured plant traits in Fairytale exposed to volatiles from different emitter cultivars (Fairytale, Luhkas, or Salome).

Table S4. Overview of measured plant traits in Salome exposed to volatiles from different emitter cultivars (Salome, Luhkas, or Fairytale).

Table S5. List of detected volatile organic compounds and their presence or absence across the investigated barley cultivars.
