# Supplementary material for: Volatiles released by undamaged plants mediate the adaptive growth strategies in neighbors
Source: J Exp Bot. 2026 May 28;77(16):5124–39. doi: 10.1093/jxb/erag252 (PMC13529331; doi:10.1093/jxb/erag252)
Supplement: Supplementary file 1 [file erag252_supplementary_data.pdf]

Supplementary material

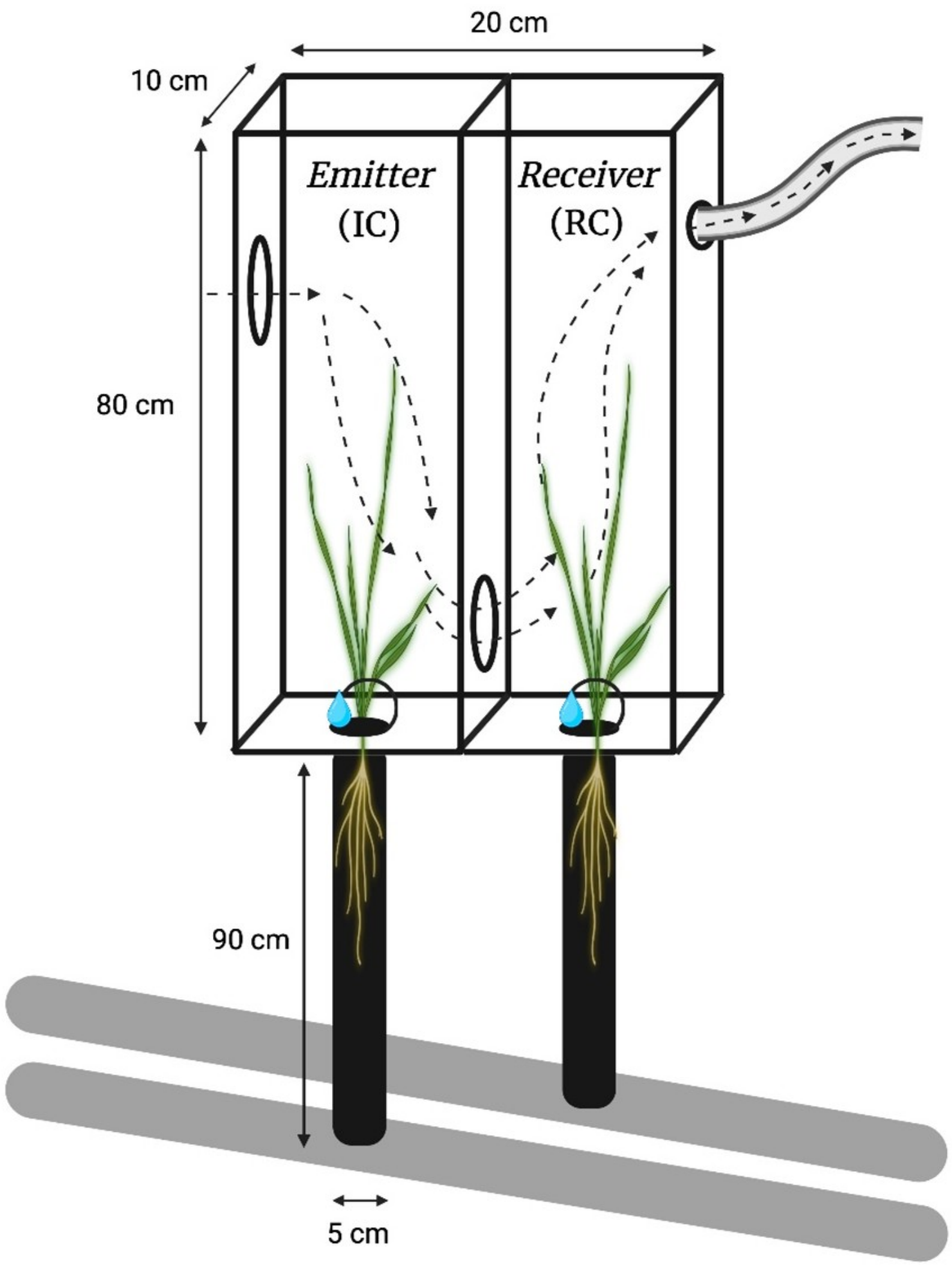

Supplementary Fig. S1. Twin-chamber volatile-exposure system. An emitter plant is grown in the inducing chamber (IC), while a receiver plant is placed in the responding chamber (RC). Air is drawn through a vent in the upper right corner of the responding chamber, creating airflow from the opening in the inducing chamber. Each chamber is equipped with an individual drip-irrigation system. Plastic tubes filled with sand are placed below the chambers to support root growth, with their lower ends connected to a drainage pipes. Dashed arrows indicate airflow and the movement of volatile organic compounds through the chambers. (Created with Biorender. Åbonde, A. (2026) https://BioRender.com/bpqddqx)

Supplementary Table S1. Leaf, stem and root biomass (mean ± SE) in Fairytale, exposed to volatiles from different emitter cultivars (Fairytale, Luhkas, or Salome): Fairytale exposed to Fairytale (FeF), Fairytale exposed to Luhkas (FeL), Fairytale exposed to Salome (FeS). Columns represent treatment means with superscript letters (a, b, c) indicating statistically significant differences among groups ($p < 0.05$, Tukey's HSD). Statistical comparisons are based on two-way ANOVA with treatment and block as fixed factors.

	Emitters			Receivers		
Biomass (g)	Fairytale	Luhkas	Salome	FeF	FeL	FeS
Leaf	$0{,}145^{a}$ ± 0,048	$0{,}188^{b}$ ± 0,04	$0{,}235^{c}$ ± 0,032	$0{,}169^{a}$ ± 0,032	$0{,}182^{a}$ ± 0,026	$0{,}192^{a}$ ± 0,018
Stem	$0{,}08^{a}$ ± 0,034	$0{,}112^{b}$ ± 0,034	$0{,}135^{b}$ ± 0,028	$0{,}099^{a}$ ± 0,025	$0{,}1^{ab}$ ± 0,016	$0{,}117^{b}$ ± 0,022
Root	$0{,}038^{a}$ ± 0,011	$0{,}05^{b}$ ± 0,01	$0{,}055^{b}$ ± 0,008	$0{,}046^{a}$ ± 0,01	$0{,}047^{ab}$ ± 0,007	$0{,}053^{b}$ ± 0,007

Supplementary Table S2. Leaf, stem and root biomass (mean ± SE) in Salome, exposed to volatiles from different emitter cultivars (Salome, Luhkas, or Fairytale): Salome exposed to Salome (SeS), Salome exposed to Luhkas (SeL), Salome exposed to Fairytale (SeF). Columns represent treatment means with superscript letters (a, b, c) indicating statistically significant differences among groups ($p < 0.05$, Tukey's HSD). Statistical comparisons are based on two-way ANOVA with treatment and block as fixed factors.

	Emitters			Receivers		
Biomass (g)	Fairytale	Luhkas	Salome	SeF	SeL	SeS
Leaf	$0{,}094^{a}$ ± 0,009	$0{,}133^{ab}$ ± 0,01	$0{,}166^{b}$ ± 0,016	$0{,}152^{a}$ ± 0,004	$0{,}169^{ab}$ ± 0,01	$0{,}195^{b}$ ± 0,011
Stem	$0{,}059^{a}$ ± 0,005	$0{,}094^{b}$ ± 0,008	$0{,}097^{b}$ ± 0,01	$0{,}089^{a}$ ± 0,005	$0{,}095^{a}$ ± 0,004	$0{,}11^{b}$ ± 0,006
Root	$0{,}037^{a}$ ± 0,004	$0{,}053^{b}$ ± 0,004	$0{,}067^{b}$ ± 0,009	$0{,}054^{a}$ ± 0,003	$0{,}065^{b}$ ± 0,003	$0{,}068^{b}$ ± 0,005

Supplementary Table S3. Overview of measured plant traits (mean ± SE) in Fairytale, exposed to volatiles from different emitter cultivars (Fairytale, Luhkas, or Salome): Fairytale exposed to Fairytale (FeF), Fairytale exposed to Luhkas (FeL), Fairytale exposed to Salome (FeS). Columns represent treatment means with superscript letters (a, b, c) indicating statistically significant differences among groups ($p < 0.05$, Tukey's HSD). Statistical comparisons are based on two-way ANOVA with treatment and block as fixed factors.

	Emitters			Receivers		
Plant trait	Fairytale	Luhkas	Salome	FeF	FeL	FeS
Leaf mass fraction (g/g)	0.55^{a} ± 0.009	0.54^{a} ± 0.007	0.55^{a} ± 0.008	0.54^{a} ± 0.01	0.55^{a} ± 0.01	0.53^{a} ± 0.01
Shoot mass fraction (g/g)	0.3^{a} ± 0.009	0.32^{a} ± 0.008	0.32^{a} ± 0.008	0.31^{a} ± 0.01	0.3^{a} ± 0.01	0.32^{a} ± 0.01
Root mass fraction (g/g)	0.15^{a} ± 0.008	0.14^{a} ± 0.003	0.13^{a} ± 0.004	0.15^{a} ± 0.004	0.14^{a} ± 0.003	0.15^{a} ± 0.004
Shoot-to-root ratio	5.96^{a} ± 0.25	5.96^{a} ± 0.16	6.78^{b} ± 0.21	5.95^{a} ± 0.18	6.04^{a} ± 0.15	5.94^{a} ± 0.19
Leaf surface area (cm^2)	57^{a} ± 4.8	75^{b} ± 4.2	104^{c} ± 3.4	69^{a} ± 3.3	72^{a} ± 2.8	76^{a} ± 2.2
Specific leaf area (cm^2g^{-1})	399^{a} ± 10.5	445^{a} ± 5.1	398^{a} ± 30.1	407^{a} ± 4.2	394^{a} ± 4.9	398^{a} ± 8.2
Plant height (cm)	46.7^{a} ± 1.9	48.6^{a} ± 1.2	50.4^{a} ± 0.7	45.6^{a} ± 1.6	48.7^{a} ± 1.4	50.1^{a} ± 0.9
Stem height (cm)	14.2^{a} ± 0.64	16^{a} ± 0.7	15.2^{a} ± 0.44	14.1^{a} ± 0.6	14.8^{a} ± 0.5	15.9^{a} ± 0.5
Root length (cm)	1007^{a} ± 67	1214^{b} ± 56.9	1394^{c} ± 40.1	1230^{a} ± 55.3	1252^{a} ± 45	1364^{a} ± 34.9
Average root Ø (cm)	0.32^{a} ± 0.003	0.33^{a} ± 0.008	0.33^{a} ± 0.005	0.33^{a} ± 0.004	0.34^{a} ± 0.004	0.33^{a} ± 0.005

Supplementary Table S4. Overview of measured plant traits (mean ± SE) in Salome, exposed to volatiles from different emitter cultivars (Salome, Luhkas, or Fairytale): Salome exposed to Salome (SeS), Salome exposed to Luhkas (SeL), Salome exposed to Fairytale (SeF). Columns represent treatment means with superscript letters (a, b, c) indicating statistically significant differences among groups ($p < 0.05$, Tukey's HSD). Statistical comparisons are based on two-way ANOVA with treatment and block as fixed factors.

	Emitters			Receivers		
Plant trait	Salome	Luhkas	Fairytale	SeS	SeL	SeF
Leaf mass fraction (g/g)	0.51^{a} ± 0.012	0.48^{b} ± 0.009	0.49^{ab} ± 0.007	0.52^{a} ± 0.014	0.51^{a} ± 0.013	0.52^{a} ± 0.006
Shoot mass fraction (g/g)	0.29^{a} ± 0.005	0.34^{b} ± 0.007	0.31^{ab} ± 0.009	0.3^{a} ± 0.009	0.29^{a} ± 0.009	0.3^{a} ± 0.009
Root mass fraction (g/g)	0.2^{a} ± 0.01	0.19^{a} ± 0.007	0.19^{a} ± 0.008	0.18^{a} ± 0.012	0.2^{a} ± 0.007	0.18^{a} ± 0.007
Shoot-to-root ratio	4.12^{a} ± 0.22	4.48^{a} ± 0.21	4.22^{a} ± 0.2	4.75^{a} ± 0.41	4.13^{a} ± 0.2	4.61^{a} ± 0.32
Leaf surface area (cm^2)	57.8^{a} ± 3.03	$44..5^{b}$ ± 3.25	32.2^{c} ± 2.23	70.9^{a} ± 2.8	64.3^{ab} ± 2.95	58.7^{b} ± 1.08
Specific leaf area (cm^2g^{-1})	371^{a} ± 22.7	339^{b} ± 14.7	366^{ab} ± 16.9	370^{a} ± 13.6	385^{a} ± 11.7	388^{a} ± 10.2
Plant height (cm)	47.2^{a} ± 0.42	48.1^{a} ± 0.67	46.5^{a} ± 0.55	50.5^{a} ± 1.02	46.4^{b} ± 0.99	47.4^{b} ± 1.02
Stem height (cm)	13.7^{a} ± 0.67	15.2^{a} ± 0.55	15.1^{a} ± 0.38	14.5^{a} ± 0.42	12.1^{b} ± 0.42	13.5^{b} ± 0.42
Root length (cm)	1345^{a} ± 75.8	1288^{a} ± 55.7	1032^{b} ± 69.9	1719^{a} ± 81.3	1490^{b} ± 62.4	1442^{b} ± 67.2
Average root Ø (cm)	0.33^{a} ± 0.012	0.31^{a} ± 0.014	0.31^{a} ± 0.016	0.31^{a} ± 0.009	0.33^{a} ± 0.007	0.32^{a} ± 0.009

Supplementary Table S5. List of detected volatile organic compounds (VOCs) and their presence (1) or absence (0) across the investigated barley cultivars (n = 9–10 per cultivar). VOCs were collected from the enclosed upper part of potted plants and reflect the complete exposure environment, including potential contributions from plant material, soil, and associated microorganisms. Compounds indicated in bold with * were confirmed by authentic reference standards. Tentatively identified compounds were assigned based on comparison with the NIST mass spectral library and reported retention indices (RI) from the NIST WebBook for non-polar HP-1 or comparable DB-1/OV-1 columns, unless otherwise indicated. All unknown compounds are labelled according to their corresponding RI values.

**Compound**	**Fairytale**	**Luhkas**	**Salome**	**RI**	**RI WebBook**

(E)-2-decenal	1	1	1	1230	1232
**(E)-2-hexenal***	1	1	0	823	-
(Z)-3-hexen-1-ol	1	1	0	834	836
**(Z)-3-hexen-1-yl acetate***	1	1	1	983	-
trimethylbenzene isomer	1	1	1	972	-
1-decanol	1	1	1	1250	1263
1-ethyl-3-methylbenzene	1	1	0	941	945
1-methyl-2-(1-methylethyl)benzene	1	1	1	1002	1009
1-methylnaphthalene	1	1	1	1257	1271
1-octanol	1	1	1	1055	1059
**1-octen-3-ol***	1	0	1	965	-
1-undecene	0	0	1	1083	1088
2,3-butanediol	1	0	0	799	824
2,4-bis(1,1-dimethylethyl)phenol	0	0	1	1485	NA (1509 on HP5)
2,6,10,14-tetramethylhexadecane	1	1	1	1817	1811
2,6-dimethyl-4-heptanone	1	1	0	952	956
2,6-dimethylundecane	1	1	0	1213	1215
2,6-di-tert-butyl-p-benzoquinone	1	1	1	1434	1440
2-ethyl-1-hexanol	1	1	1	1010	1012
2-ethylhexanal	1	0	0	932	933
2-methyldodecane	1	1	0	1262	1264
2-methylnaphthalene	1	1	1	1271	1268
2-methylpentadecane	1	1	1	1565	1566
2-methyltridecane	1	1	1	1362	1366
2-phenylethanol	1	0	0	1078	1082
3-methylpentadecane	1	1	1	1570	1574
3-methyltridecane	1	1	1	1365	1373
6,10,14-trimethyl-2-pentadecanone	1	1	1	1828	1809
8-heptadecene	0	1	0	1675	NA
**Acetophenone***	1	1	1	1025	-
Benzaldehyde	1	1	1	920	924
Benzoic acid	0	1	0	1154	1159
Benzothiazole	1	1	1	1176	1180
**Benzyl alcohol***	1	1	1	1003	-
Benzyl nitrile	1	1	1	1084	1095
Decanal	1	1	1	1182	1184
**Decane***	1	1	0	1000	-
**Dodecane***	1	1	1	1200	-
**Eicosane***	1	1	1	2004	-
**Heptadecane***	0	0	1	1700	-
Heptanal	1	1	1	874	876
**Hexadecane***	1	1	1	1600	-

Limonene	1	1	1	1017	1022
**Linalool***	1	1	1	1081	-
**Linalool oxide***	0	0	1	1067	-
**Methyl salicylate***	1	1	1	1162	-
Naphthalene	1	1	1	1185	1190
**Nonadecane***	1	1	1	1900	-
Nonanal	1	1	1	1080	1078
**Nonane***	1	1	0	900	-
Octanal	1	1	1	979	984
**Octane***	1	1	1	800	-
**Pentadecane***	1	1	1	1500	-
Styrene	1	1	1	864	872
**Tetradecane***	1	1	1	1394	-
**Tridecane***	1	1	1	1294	-
**Undecane***	1	1	1	1099	-
**β-caryophyllene***	1	1	0	864	-
**β-ocimene***	0	1	1	1034	-
δ-valerolactone	1	1	1	994	991
RI 846	1	1	1		
RI 855	1	1	1		
RI 860	1	1	1		
RI 911	1	1	1		
RI 955	1	1	1		
RI 967	1	1	1		
RI 981	1	1	1		
RI 994	1	1	0		
RI 1054	0	0	1		
RI 1059	1	1	0		
RI 1089	1	1	1		
RI 1093	1	1	1		
RI 1119	1	1	1		
RI 1131	1	1	1		
RI 1157	0	0	1		
RI 1160	1	1	1		
RI 1201	0	0	1		
RI 1224	1	1	1		
Ri 1245	1	1	1		
RI 1267	0	0	1		
RI 1269	1	1	0		
RI 1282	1	1	1		
RI 1300	1	1	1		
RI 1313	1	1	0		
RI 1314	0	1	0		
RI 1317	1	1	1		
RI 1384	1	1	1		
RI 1389	1	1	1		

RI 1391	1	1	0
RI 1446	1	0	0
RI 1458	1	1	0
RI 1461	1	1	1
RI 1546	1	1	1
RI 1571	1	1	1
RI 1589	1	1	1
RI 1649	1	1	1
RI 1669	0	1	1
RI 1677	0	1	1
RI 1688	0	1	1
RI 1692	0	1	1
RI 1694	0	1	1
RI 1698	1	1	1
RI 1722	0	1	1
RI 1731	0	1	1
RI 1743	1	1	1
RI 1779	1	1	1
RI 1803	1	1	1
RI 1805	1	1	1
RI 1819	1	1	1
RI 1947	1	0	0
RI 1947	1	0	1
RI 1974	1	1	1
RI 2070	1	1	1
RI 2169	0	1	1
RI 2291	0	1	0
